# Efficacy of a multi-component exercise programme and nutritional supplementation on musculoskeletal health in men treated with androgen deprivation therapy for prostate cancer (IMPACT): study protocol of a randomised controlled trial

**DOI:** 10.1186/s13063-017-2185-z

**Published:** 2017-10-03

**Authors:** Patrick J. Owen, Robin M. Daly, Patricia M. Livingston, Niamh L. Mundell, Jack Dalla Via, Jeremy L. Millar, Steve F. Fraser

**Affiliations:** 1 Deakin University, Institute for Physical Activity and Nutrition (IPAN), School of Exercise and Nutrition Sciences, Geelong, Australia; 2 Deakin University, Faculty of Health, Geelong, Australia; 3 Alfred Health Radiation Oncology, The Alfred, Melbourne, Australia

**Keywords:** Prostate cancer, Androgen deprivation therapy, Bone mineral density, Body composition, Cardiometabolic health, Exercise training, Protein, Calcium, Vitamin D, Randomised controlled trial

## Abstract

**Background:**

Prostate cancer is the most commonly diagnosed cancer in men in developed countries. Androgen deprivation therapy (ADT) is a systemic treatment shown to increase survival in selected patients with prostate cancer. The use of ADT continues to increase for all stages and grades of prostate cancer despite known treatment-induced adverse effects. The primary aim of this study is to examine the efficacy of a targeted, multi-component resistance and impact-loading exercise programme together with a daily protein-, calcium- and vitamin D-enriched supplement on bone health in men treated with ADT for prostate cancer. Secondary aims are to determine the effects of this intervention on measures of total body and regional body composition, cardiometabolic risk, inflammatory markers, health-related quality of life and cognitive function.

**Methods:**

This study is a two-arm randomised controlled trial. Men currently treated with ADT for prostate cancer will be randomised to either a 52-week, community-based, exercise training and nutritional supplementation intervention (*n* = 51) or usual care control (*n* = 51). Participants will be assessed at baseline, 26 weeks and 52 weeks for all measures. The primary outcome measures are proximal femur and lumbar spine areal bone mineral density (BMD). Secondary outcomes comprise: changes in tibial and radial bone structure and strength, total body and regional body composition, muscle strength and function, as well as cardiometabolic health, catabolic/inflammatory and anabolic/anti-inflammatory cytokines, health-related quality of life and cognitive function.

**Discussion:**

This study investigates whether a multi-component intervention incorporating a targeted bone and muscle-loading programme in combination with a protein-, calcium- and vitamin D-enriched supplement can ameliorate multiple adverse effects of ADT when compared to usual care. The results will contribute to the development of exercise training and nutrition guidelines for optimising overall health in men treated with ADT for prostate cancer.

**Trial registration:**

Australia New Zealand Clinical Trial Registry (ANZCTR), ID: ACTRN12614000317695. Registered on 25 march 2014.

**Electronic supplementary material:**

The online version of this article (doi:10.1186/s13063-017-2185-z) contains supplementary material, which is available to authorized users.

## Background

In Australia, as in other developed countries, prostate cancer (PCa) is the most commonly diagnosed cancer in men [1]. Approximately 1.1 million new cases of PCa are diagnosed worldwide each year [1]. In Australia, a 2014 report estimated that approximately 13% of cancer-related deaths in men were attributed to PCa, which ranks second to lung cancer as the most common cause of cancer-related death in men [2]. Due to medical advancements in both the screening and treatment of PCa, 5-year relative survival rates of all PCa cases are now approaching 100% internationally [3], and thus these patients are living longer, but are susceptible to potential age-related and treatment-related declines in health. Therefore, the clinical paradigm is shifting to an increased focus on improving the quality of years lived.

Various modalities of androgen deprivation therapy (ADT), including those administered as neoadjuvant or adjuvant therapies with other treatments, are commonly used to treat PCa as it improves overall survival, particularly in men with advanced PCa [4–7]. However, the evidence that ADT prolongs survival in men with localised PCa remains limited and, therefore, debated [8, 9]. Despite this lack of evidence, the use of ADT continues to increase for all stages and grades of PCa [10]. It was estimated that approximately 23,500 Australian men received pharmacological ADT via gonadotropin-releasing hormone (GnRH) agonists in 2008–2009 [11]. After repeating this analysis for 2013–2014, we estimated that approximately 25,500 men are currently receiving ADT via GnRH agonists [12]. Thus, ADT remains the most common cause for severe hypogonadism in men in Australia.

Despite benefits in overall survival in appropriately selected men, the hypogonadism induced via ADT has been associated with numerous adverse effects, particularly with regard to musculoskeletal and cardiometabolic health. It has been reported that within the first 3 to 12 months of ADT, men experience a rapid and dramatic loss in bone mass (up to 11%) and concomitant losses in muscle mass (2–5%) [13–17] which may explain the observed 23–65% increased fracture risk in this group [18–20]. There is also a profound gain (up to 15%) in fat mass (FM) which likely contributes to the 10–45% reported increased risk of diabetes, coronary heart disease, myocardial infarction and sudden cardiac death [13, 17, 21–25]. Thus, there is a need to develop safe and effective interventions to manage the multiple treatment-induced adverse effects of ADT in men with PCa.

Exercise training has been strongly suggested as a viable intervention to ameliorate many of the adverse effects of ADT [17, 26–28]. In terms of musculoskeletal health outcomes, a number of interventions have shown that progressive resistance training (PRT) can increase muscle strength [29–33], muscle endurance [30, 34] and balance [30] in men treated with ADT. While there is also some evidence that PRT can improve or preserve lean body mass (LBM) [30, 32, 33, 35], a systematic review found that there was inconclusive findings on the effects of exercise training in men treated with ADT on bone health [26]. To date, only four studies have examined the efficacy of exercise training on areal bone mineral density (aBMD) in ADT-treated men, three of which were conducted over interventional periods of no longer than 20 weeks and observed no benefit [32, 36, 37]. This is perhaps not unexpected given that the typical bone remodelling cycle and new steady state that is measurable takes 6–8 months to complete, and thus it is unlikely that any true physiological skeletal adaptations would occur prior to this period. However, a recent 12-month randomised controlled trial (RCT) also reported no marked skeletal benefits of a targeted PRT and impact-loading exercise programme in men treated with ADT [36]. This may be due to a number of factors. First, the sample size of approximately 25 per group was below the target sample which may have limited the power to detect any between-group differences. Second, the impact exercise programme was non-progressive and only consisted of two-footed jumps (plus 10% body weight), and thus did not incorporate diverse loading patterns which are known to be important for bone remodelling. Third, the mean calcium intake was < 750 mg/day, well below the recommended dietary intake of 1200 mg/day. There is some evidence that insufficient calcium intakes can attenuate the skeletal responses to exercise in older adults [38]. Finally, this study was limited to an assessment of aBMD by dual-energy X-ray absorptiometry (DXA), which provides no information on other determinants of bone strength (e.g. trabecular volumetric BMD (vBMD), cortical structure), which may change following exercise training, independent of aBMD. This is important because the findings from a prospective study using high-resolution, peripheral, quantitative computed tomography (pQCT) found that treatment with ADT for 12 months was associated with losses of up to 11.3% in cortical and 3.5% in trabecular vBMD, which are two- to three-fold greater than changes observed in DXA aBMD [14]. In healthy older men, the findings from an 18-month RCT found that a targeted and progressive multi-component exercise programme incorporating PRT and a diverse range of weight-bearing, impact activities was safe and effective for improving lumbar spine trabecular vBMD and femoral neck aBMD, cross-sectional area and strength as well as muscle strength, mass and size [39]. Further long-term RCTs are required to examine whether similar well-designed and progressive multi-component exercise programmes in combination with adequate nutrition can ameliorate bone and muscle loss in men treated with ADT for PCa.

Clinical guidelines for optimising bone health in men on ADT recommend calcium and vitamin D treatment [11]. While direct evidence from RCTs to support their role in preventing bone loss in this group is lacking, a review based on secondary analyses of clinical trials reported that treatment with 500–1000 mg/day of calcium and 200–500 IU/day of vitamin D in ADT-treated men was inadequate to prevent bone loss [40]. In healthy older adults, there is level-1 evidence that calcium (>1000 mg/day) plus vitamin D (> 800 IU/day) can reduce fracture risk [41]. Studies have also shown that 2000 IU/day of vitamin D can improve muscle strength and function [41, 42], and that daily consumption of calcium-vitamin D fortified milk for 2 years prevented aBMD loss at multiple sites in healthy men aged older than 50 years [43]. While a systematic review and meta-analysis of prospective studies reported that increased dairy or calcium intake was associated with an increased risk of PCa, no association was observed with supplemental or non-dairy calcium, which suggest that other components of dairy rather than fat and calcium may increase PCa risk [44]. In line with these mixed findings, supplementation at doses < 1500 mg/day have been shown to either have no influence on PCa progression [45], reduce PCa risk [46] or decrease PSA velocity [47]. In addition, in men diagnosed with PCa, total milk/dairy intake after diagnosis was not associated with a greater risk of lethal PCa, with evidence of a decreased risk in those with a high intake of low-fat dairy [48].

There is also evidence that increased dietary protein in older adults can have favourable effects on both health, particularly when combined with calcium and vitamin D at recommended levels [38]. This has been attributed to several factors: a higher protein intake can increase serum insulin-like growth factor-1 (IGF-1), enhance intestinal calcium absorption, suppress parathyroid hormone levels as well as increase muscle mass and strength, which may improve bone health via increased loading on bone [49]. There is also evidence in older adults without PCa that higher protein intakes (> 1.2 g/kg) can enhance weight loss, offset muscle loss, improve lipid profiles and insulin sensitivity [50]. While this is not a universal finding, this could be explained by the modest level of protein intake achieved in some studies and/or the type (quality) of protein consumed. Currently there are no guidelines with regard to dietary protein for men with PCa using ADT.

It is well known that PRT stimulates muscle protein synthesis (MPS), but in a fasted state it also accelerates muscle protein breakdown (MPB) [51]. Ingestion of a high-quality, rapidly digested, protein-rich source, such as whey-protein, post-exercise can attenuate the increase in MPB and stimulate MPS to enhance the anabolic benefits of PRT [51]. Thus, combining PRT with whey-protein ingestion may represent an optimal strategy to enhance muscle hypertrophy. While questions still remain in terms of the optimal dose of protein needed to elicit a synergistic response with PRT, emerging evidence indicates that 20–40 g of high-quality protein be consumed early after a bout of PRT to enhance muscle mass and/or strength [51]. Moreover, Hanson et al. [52] recently demonstrated that consumption of 40 g of whey-protein alone, or following a single session of unilateral knee extension exercises, increased MPS beyond basal rates in men treated with ADT; albeit these increments were suboptimal when compared to healthy, age-matched men. However, a critical, but as yet unanswered, question is whether ingestion of a whey-protein-, vitamin D- and calcium-enriched drink with PRT can improve the multiple musculoskeletal health outcomes in ADT-treated men.

Therefore, the primary aim of this 12-month RCT is to determine whether a multi-component exercise programme targeting muscle and bone health combined with a protein-, calcium- and vitamin D-enriched drink can enhance hip and lumbar spine aBMD in men with PCa currently treated with ADT. The secondary aims are to examine the effects of the intervention on tibial and radial bone structure and strength, total body and regional body composition, muscle strength and function, as well as cardiometabolic health, catabolic/inflammatory and anabolic/anti-inflammatory cytokines, health-related quality of life (HR-QoL) and cognitive function.

## Methods

### Study design

This will be a 52-week, single-blinded, two-arm RCT consisting of a supervised and structured multi-component exercise programme in combination with a daily nutritional supplement. Men treated with ADT will be randomised to either an intervention consisting of a multi-component exercise programme and a daily protein-, calcium- and vitamin D-enriched supplement (*n* = 51) or a usual care control group (*n* = 51). Men allocated to usual care will not receive a placebo drink as typical ingredients used to develop a placebo drink (e.g. maltodextrin as a food additive) have the potential to alter blood glucose levels and muscle mass. A factorial 2 × 2 trial will not be conducted for several reasons: (1) the large sample size that would be required to detect an interaction and (2) findings from previous research in older adults which indicates that the benefits of exercise training on musculoskeletal health require adequate nutrition (e.g. dietary calcium > 1000 mg/day) [38]. This study will not evaluate whether the combination of protein, calcium and vitamin D with exercise training is more effective than either of these treatments alone, but will determine if combining the two approaches is effective for improving multiple health outcomes. This trial will be managed by the Institute for Physical Activity and Nutrition at Deakin University. The study has been approved by the Human Research Ethic Committees at Deakin University (HREC 2013-184) and Alfred Health (Project No: 455/15) and is registered with the Australian and New Zealand Clinical Trials Registry (ACTRN12614000317695). A summary of trial resgistration data and compliance with SPIRIT guidelines are shown in Additional files 1 and 2, respectively.

### Participants

Eligible participants will be men aged 50–85 years who are currently treated with ADT for histologically diagnosed PCa. Treatment with ADT must be pharmacological (surgical orchiectomy excluded) and administered for longer than 12 weeks at enrolment. Participants will be excluded if they do not have the ability to complete surveys in the English language, have any disorder known to affect bone, calcium or vitamin D metabolism (other than hypogonadism), are currently receiving pharmacological intervention known to affect bone metabolism (other than ADT), have supplemented with protein, calcium (> 600 mg/day) or vitamin D (> 1000 IU/day) in the past 3 months, have undertaken PRT (more than one session/week) or regular weight-bearing, impact exercise (> 150 min/week) in the past 3 months, are current smokers, have a weight greater than 159 kg, have plans to travel for longer than 6 weeks continuously within the following 52 weeks or have any absolute contraindications to exercise training (e.g. musculoskeletal, cardiovascular or neurological disorders) according to the American College of Sports Medicine guidelines [53].

### Recruitment and screening

Participants will be recruited via direct referral from clinicians across various hospitals, private practices and health networks in combination with promotion via PCa support groups and advertisements in state/local newspapers within Melbourne and surrounding areas in Victoria, Australia. The direct referral process will include mass mail-outs to potential participants and face-to-face discussions during regular consultations with physicians/specialists. PCa support groups will be initially contacted via email with information regarding the study, with an option to provide a brief presentation to groups interested. All potential participants will be screened initially via telephone to determine their eligibility based on the criteria outlined above. All eligible participants will be required to obtain medical approval from their physician and provide informed consent before participating in the study.

### Randomisation and blinding

**Fig. 1:**
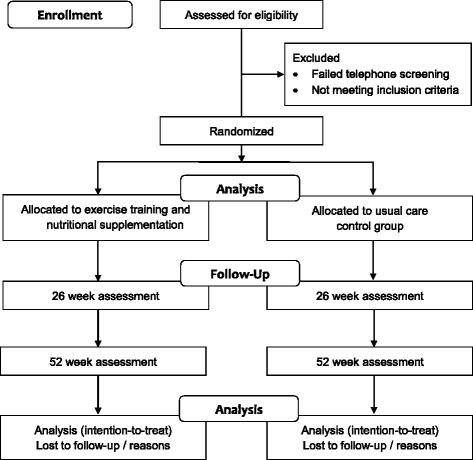
Consolidated Standards of Reporting Trials (CONSORT) diagram

Randomisation will be at the level of the individual participant following baseline testing, stratified by age (< 65 or ≥ 65 years) and Body Mass Index (< 30 or ≥ 30 kg/m^2^), using a computer-generated, random number sequence by an independent researcher. Due to limited funding, neither the participants nor researchers involved in the testing will be blinded to the group allocation, with the exception of the DXA and pQCT scans which will be assessed independently. A flow diagram of the study protocol is outlined in Fig. 1.

### Intervention group

#### Multi-component exercise programme

The 52-week exercise programme will consist of two gym-based sessions and one home-based session per week. During the first 26 weeks, the two gym-based sessions will be supervised by an accredited exercise physiologist (tertiary-trained exercise professional) in a community-based health and fitness facility. For the final 26 weeks, only one gym-based session will be supervised. All home-based sessions will be unsupervised and performed in the participant’s own time. All intervention-group participants will be provided with a yearly membership at a community-based health and fitness centre and will have the opportunity to complete their home-based sessions in this setting at their own discretion.

The exercise training programme is a multi-component programme that will follow key training principles of specificity and progressive overload and will be individually tailored to the participant’s health and functional status. The programme will follow similar exercise prescription guidelines in terms of the structure, type and dose (frequency, sets, repetitions, intensity) of training to our previous ‘*Osteo-cise*: Strong Bones for Life’ community-based osteoporosis prevention exercise programme in healthy older adults in which we observed significant improvements in musculoskeletal health and function (Table 1) [39, 54, 55]. To ensure progressive overload and promote a sense of achievement for the men, the 52-week programme will be divided into five mesocycles (training phases). The first mesocycle (4 weeks – adoption phase) will focus on orientation to the exercise programme, with an emphasis on correct technique. The four subsequent 12-weekly mesocycles will focus on progression through three 4-weekly microcycles (preparatory phase, challenge phase and consolidation phase). Each gym-based session (approximately 60 min) will consist of a warm-up and cool-down and a combination of aerobic training, PRT, weight-bearing, impact activities and challenging balance/functional exercises. More specific details about each training component are provided below.

**Table 1:** Structure of the exercise programme and training doses for 52 weeks

Phase			Aerobic training		Progressive resistance training					Weight-bearing, impact training			Functional and core stabilisation training	
Microcycle	Week	Mesocycle	Duration	%HR_MAX_	Sets	Reps	RPE^1^	Speed^2^	Rest	Sets	Reps	Intensity^3^	Sets	Duration/reps
#1	1–4	Orientation	15–25 min	55–75%	2	12–15	3–4	Slow	1–2 min	3	10–20	Low	2	Balance exercises, 30–60 s or until fatigue Core stabilisation exercises, 10–15 reps or up to 50 for abdominal based exercises
#2	5–8	Preparatory	15–25 min	60–75%	2	10–-15	3–4	Slow	1–2 min	3	10–-20	Low to moderate	2	
	9–12	Challenge	15–25 min	60–75%	2	8–12	5–8	Rapid	2 min	3	10–20		2	
	13–16	Consolidation	15–25 min	60–75%	2	8–12	5-8	Rapid	2 min	3	10–20		2	
#3	17–20	Preparatory	15–25 min	60–75%	2	10–15	3–4	Slow	1–2 min	3	10–20	Moderate	2	
	21–24	Challenge	15–25 min	60–75%	2	8–12	5–8	Rapid	2 min	3	10–20		2	
	25–28	Consolidation	15–25 min	60–75%	2	8–12	5–8	Rapid	2 min	3	10–20		2	
#4	29–32	Preparatory	15–25 min	60–75%	2	10–15	3–4	Slow	1–2 min	3	10–20	Moderate to high	2	
	33–36	Challenge	15–25 min	60–75%	2	8–12	5–8	Rapid	2 min	3	10–20		2	
	37–40	Consolidation	15–25 min	60–75%	2	8–12	5–8	Rapid	2 min	3	10–20		2	
#5	41–44	Preparatory	15–25 min	60–75%	2	10–15	3–4	Slow	1–2 min	3	10–20	High	2	
	45–48	Challenge	15–25 min	60–75%	2	8–12	5–8	Rapid	2 min	3	10–20		2	
	49–52	Consolidation	15–25 min	60–75%	2	8–12	5–8	Rapid	2 min	3	10–20		2	

Adapted from Gianoudis et al. ^1^RPE: Rating of Perceived Exertion (1–10); ^2^Speed: slow (2–3 sec concentric and 2–3-s eccentric contractions), rapid (rapid concentric and 2–3-s eccentric contractions); ^3^Intensity: low (ground reaction force (GRF) ~ 1–3 × body weight), moderate (GRF ~ 3–6 × body weight), high (GRF ~ 6–9 × body weight)

#### Aerobic training

For the aerobic exercise, participants will be prescribed 15 to 25 min of continuous aerobic training at 55–75% of predicted heart rate maximum via modalities such as stationary cycling, treadmill walking or rowing. The type of aerobic exercise prescribed will be based on each participant’s preference and functional capabilities.

#### Progressive resistance training

For PRT, participants will be prescribed five or six targeted hip, spine and forearm resistance exercises using machine and free weights (two sets of 12–15 repetitions at moderate intensity (3-4 on the 10-point Rating of Perceived Exertion (RPE) scale) for each exercise for the first 4-8 weeks, progressing to two sets of 8–12 repetitions at moderate to hard intensity (4–6 on the RPE scale) thereafter; resistance will be increased as tolerated to maintain intensity and ensure progressive overload). During the first mesocycle, five resistance exercises will be available to choose from for each training session (leg press, bench press, one hip-targeted, one-spine targeted and one upper body exercise). New resistance exercises will be introduced at the start of each subsequent mesocycle (three hip-targeted, three spine-targeted and one upper- body-targeted). Varying combinations of the nine resistance exercises in each of these mesocycles will be prescribed each session. After the initial 8 weeks of training, participants will be progressively taught to perform the concentric (lifting) phase of all lower body exercises at high velocity. This is designed to improve movement speed and muscle power. When possible, participants will perform resistance exercises standing with the aim to also improve balance and physical function.

#### Weight-bearing, impact exercise

For the weight-bearing, impact-loading exercises, participants will be prescribed three exercises (interspersed amongst the PRT exercises) consisting of three sets of 10–20 repetitions that will utilise body weight as the primary load. The programme will focus on lower-limb exercises designed to load the hips. The exercises prescribed will be based on a battery that we have previously shown to be safe and effective for improving femoral neck and lumbar spine aBMD in older men [54]. This battery includes a range of exercises from low to high intensity (magnitude of peak ground reaction forces) and difficulty (easy, moderate, hard) and is divided into three broad categories: stationary movements (e.g. marching on the spot, mini-tuck jumps), forward/backwards movements (e.g. forward bench step-ups, drop jumps) and lateral movements (e.g. side-to-side jumping). Training intensity will be increased progressively by increasing the height of jumps, by adding additional weight, increasing the rate of impact-loading or multi-directional movement patterns (e.g. diagonal loads).

#### Challenging balance/functional exercise

Challenging balance and functional exercises will include a range of static and dynamic exercises, including fit ball exercises (e.g. sitting on fit-ball with knee extension), standing balance activities (e.g. single-leg standing) and dynamic functional tasks (e.g. heel-to-toe walking). Participants will be prescribed two exercises per session and asked to complete two sets of 30–60 s or for a given number of repetitions. To ensure progression, participants will be prescribed a new exercise or advance to a more challenging level (e.g. closing eyes, reducing base of support, confounding visual fixation, changing centre of mass or adding a second manual or cognitive task) once they have mastered the previous exercise (e.g. it is no longer challenging).

#### Core stabilisation exercise

Key muscle groups known to help maintain good posture, improve pelvic floor function and trunk stability (e.g. rotator cuff, scapular stabilisers, transverse abdominis, pelvic floor muscles) will be targeted with these exercises, which are important for balance and muscular performance [56]. Participants will be prescribed two exercises per session with light to moderate resistance in two sets of 10 to 15 repetitions. Of note, abdominal-based exercises will be prescribed in two sets of up to 50 repetitions.

#### Home-based exercise programme

The home-based exercise programme will consist of similar types of exercise to the gym-based sessions, but body weight and resistive tubing (provided to participants) will be used for resistance exercises. This will provide participants with knowledge and experience of ways to exercise relatively inexpensively in a home-based setting following the completion of the study. The home-based programme will be explained and demonstrated to the participants during the initial gym-based sessions and instructions will be provided. Three resistance exercises, three weight-bearing, impact-loading exercises and three functional exercises following the same training principles as gym-based sessions will be prescribed for each home-based session. The home-based programme will be progressed and new exercises will be introduced once the previous exercises are no longer challenging for the participant.

#### Nutritional supplement

The nutritional supplement will consist of a daily whey-protein-, calcium- and vitamin D-enriched drink (powder) combined with a single vitamin D tablet. Participants will be provided with 6-monthly supplies of powder in single-use sachets and tablets. Each sachet is to be mixed with 150 ml of water and shaken vigorously in a ‘shake-and-take’ container (provided to participant). Each sachet of powder will contain approximately 25 g of whey-protein concentrate 80% (WPC80), containing approximately 2.4 g of leucine, 1200 mg calcium carbonate (~ 480 mg elemental calcium) and 1000 IU vitamin D (Omniblend, Campbellfield, VIC. Australia). The vitamin D tablet will contain 1000 IU (Ostelin, Macquarie Park, NSW, Australia). On the non-training days, participants will be asked to take one sachet every morning before breakfast. This is designed to try and ensure a more even protein intake throughout the day which has been reported to optimise muscle protein balance [57]. On exercise training days, participants will be encouraged to consume the sachet within 1–2 h of completing their exercise session as early post-exercise consumption of protein has been shown to promote an increase in MPS and muscle hypertrophy in older adults [58, 59].

### Usual-care control group

Participants allocated to usual care will receive ongoing care from their physician/specialist but will not receive additional education or access to exercise training or the protein-, calcium- and vitamin D-enriched nutritional supplement powder sachet. Due to current practice guidelines [40] and a pilot study we conducted in 31 men undergoing ADT for PCa in which 65% reported taking vitamin D supplementation (90% at least 1000 IU/day), all men randomised to usual care will receive 1000 IU vitamin D per day. There is no evidence that vitamin D alone (without calcium supplementation) at these doses can prevent bone loss in these men [40].

### Measurements

All measures will be collected at baseline, 26 and 52 weeks. Additionally, 24-h food recalls will also be administered at 13 and 39 weeks to monitor any habitual fluctuations in dietary factors. With the exception of blood samples, 24-h food recalls and questionnaires, all data will be collected at Deakin University. Blood samples will be collected at an external commercial pathology clinic. The 24-h food recalls will be administered via telephone. Questionnaires will be completed by participants at home prior to each testing sessions (i.e. baseline, 26 and 52 weeks) and checked by the research staff during these face-to-face testing sessions. A summary of the proposed outcome measures is shown in Table 2. All participants will undergo all measures expect those specific to treatment allocation (i.e. compliance to the intervention). All data collected will be in a continuous format, aside from the outcomes from some of the questionnaires, which will be categorical in nature.

**Table 2:** Summary of data collection

Variables	Data collection method	Data collection points		
		Baseline	26 weeks	52 weeks
Primary outcome measures				
Areal bone mineral density	DXA proximal femur and lumbar spine	x	x	x
Secondary outcome measures				
Bone structure and strength	pQCT scan at 4% and 66% radial and tibial site	x	x	x
Body composition	DXA total body and regional lean mass, fat mass and % body fat	x	x	x
	pQCT 66% radial and tibial muscle cross-sectional area and muscle density	x	x	x
Muscle strength	Chest press, leg press and seated row	x	x	x
	Grip strength	x	x	x
Functional capacity	30-s sit-to-stand	x	x	x
	Timed-up-and-go with cognitive task	x	x	x
	Four-square step test	x	x	x
	Berg balance scale	x	x	x
	Four-metre usual walk	x	x	x
	400-metre walk	x	x	x
Biochemistry and lipids	Overnight, fasted serum and plasma collection	x	x	x
Inflammatory markers	Overnight, fasted serum and plasma collection	x	x	x
Blood pressure	Automated measurement	x	x	x
Cognitive function	CogState Brief Battery computerised tests	x	x	x
	Rey Auditory Verbal Learning Test	x	x	x
	Trail Making Test (Part A and B)	x	x	x
	Digit Span Test	x	x	x
	National Adult Reading Test	x		
Health-related quality of life, fatigue and mood	Functional Assessment of Cancer Therapy-Fatigue questionnaire	x	x	x
	Functional Assessment of Cancer Therapy-Prostate questionnaire	x	x	x
	Assessment of Quality of Life 8D questionnaire	x	x	x
	Geriatric Depression Scale questionnaire	x	x	x
	Depression Anxiety and Stress Scale questionnaire	x	x	x
Additional measures				
Anthropometry	Height, weight, Body Mass Index, waist and hip circumference	x	x	x
Habitual physical activity	Community Healthy Activities Model Programme for Seniors physical activity questionnaire	x	x	x
Diet	24-h food recalls	Collected every 13 weeks		
Additional questionnaire	Demographic questionnaire	x		
	Clinical and lifestyle questionnaire	x	x	x
	Sun exposure habits and practices questionnaire	x	x	x
	Alcohol consumption questionnaire	x	x	x
	Medical questionnaire	x	x	x
	Medication questionnaire	x	x	x
	Falls questionnaire	x	x	x
Exercise programme adherence	Calculated from monthly calendars collected every 4 weeks	Collected every 4 weeks		
Supplement compliance	Calculated from supplement returned at 26 and 52 weeks		x	x
Adverse events	Calculated from monthly calendars collected every 4 weeks	Collected every 4 weeks		

*DXA* dual-energy X-ray absorptiometry, *pQCT* peripheral quantitative computed tomography

### Primary and secondary outcome measures

The primary outcome for this study will be changes in proximal femur and lumbar spine aBMD. Secondary outcomes will comprise: changes in 4% and 66% tibial and radial vBMD, bone structure and strength, total body and regional body composition, muscle strength, functional capacity, cardiometabolic health, inflammatory biomarkers, HR-QoL and cognitive function.

#### Bone mineral density

Total hip, femoral neck and lumbar spine (L1–L4) aBMD (g/cm^2^) will be assessed using DXA (Lunar Prodigy, GE Lunar Corp., Madison, WI, USA) using software version 12.30.008. The short-term coefficient of variation (CV) for aBMD measures range from 0.6 to 1.0% within our laboratory.

#### Volumetric BMD, bone geometry and strength

Proximal (66%) radial and tibial cortical vBMD (mg/cm^3^), cortical area (mm^2^), total bone area (mm^2^), medullary area (mm^2^) and polar Strength Strain Index (SSIpolar, mm^3^), and distal (4%) radial and tibial total and trabecular vBMD (mg/cm^3^), total bone area (mm^2^) and the Bone Strength Index (BSI, mg^2^/mm^4^) of the non-dominant upper limb and dominant lower limb will be assessed using pQCT (XCT 3000, Stratec Medizintechnik GmbH, Pforzheim, Germany). Briefly, tibial length will be determined by measuring from the distal edge of the medial malleolus to the medial joint cleft of the knee and radial length will be determined by measuring ulnar length from the ulnar styloid process to the olecranon. After obtaining a scout-view slice of the distal endplate of the radius or tibia, scans will be performed at the 4% and 66% sites. The slice thickness will be 1 mm and voxel size will be 0.5 mm at a scanning speed of 20 mm/s. All pQCT bone and body composition parameters will be assessed using BoneJ [60] ImageJ-plugin (rsbweb.nih.gov/ij) as previously reported [61]. Distal radial and tibial (4%) total bone area and density will be analysed based on thresholding at 169 mg/cm^3^, with trabecular density assessed using a threshold of 480 mg/cm^3^. BSI will be calculated as follows: BSI = total area multiplied by the square of total vBMD [62]. For the proximal radius and tibia (66%), the periosteal surface will be determined based on a threshold of 280 mg/cm^3^, and cortical bone a threshold of 710 mm/cm^3^. Medullary area will be calculated by subtracting cortical area from total area. SSIpolar will be determined using the bone threshold of 480 mg/cm^3^ [63]. The short-term CVs for repeated measures in a sample of healthy premenopausal women range from 0.9 to 2.2% for the 4% radius, 0.7 to 2.5% for the 4% tibial and 0.6 and 1.8% for the 66% tibial outcome measures [64].

#### Body composition

Total and regional (arms and legs) LBM (kg), FM (kg) and percentage body fat will be assessed by DXA as described above. The short-term CV for LBM and FM repeated measures range from 1.0 to 1.7% within our laboratory. Muscle and subcutaneous fat cross-sectional area (CSA) and muscle density (as a measure of intermuscular adiposity) at the proximal (66%) radius and tibia will be assessed using pQCT. Thresholds of − 40 to + 40 mg/cm^3^ hydroxyapatite density will be used for estimating subcutaneous fat CSA. Muscle CSA will be estimated by subtracting the total bone CSA and subcutaneous fat CSA from the total area of the 66% tibia or radius. The following CVs have been reported for muscle CSA (radius, 2.1 to 5.3%; tibia, 2.5 to 3.7%), muscle density (radius, 1.4 to 3.2%; tibia 0.7 to 3.2%) and subcutaneous fat CSA (radius, 2.4 to 3.2%; tibia, 6.0 to 6.3%) for repeated measures in a sample of postmenopausal women [65].

#### Muscle strength

Muscle strength will be obtained via chest press (BodySolid, Powerline Smith Machine PSM144X, Forest Park, IL, USA), leg press (Synergy Omni Leg Press S-31-OPD, Yatala, QLD, Australia) and seated row (Nautilus tower pulley system, F3ATFS, Independence, VA, USA) three-repetition maximum (3-RM) protocols [53]. The 3-RM protocols assess the maximum weight that can be lifted for three repetitions while maintaining correct form and technique and were selected rather than one-repetition maximum protocols to reduce participant exertion. Prior to attempting these protocols, participants will complete a 5-min aerobic warm-up. Thereafter, each participant will perform a warm-up set of eight to 10 repetitions with a light load. The participant will then complete six to eight repetitions at a heavier weight. The load will be increased incrementally until only three repetitions can be completed. A 2–3-min recovery period will be provided between each set. The 3-RM result will be used in a formula to calculate each participant’s one-repetition maximum [66]. The 3-RM protocol has been shown to have a test-retest intraclass correlation coefficient (ICC) of 0.97 in a sample of untrained men [67] and strongly correlates with 1-RM results [68].

Maximal muscle (grip) strength will be assessed using a digital grip-strength dynamometer (Jamar Plus Digital, Lafayette Instrument Company, IN, USA) [69]. The participant will be seated with their foreman resting on the arm of chair while maintaining a 90° angle at the elbow. Participants will be asked to squeeze the dynamometer maximally. Six trials will be completed (three with each hand alternating) with the single highest score recorded. A CV of 6.3% was reported for grip-strength repeated measures with the same device in a sample of advanced lung and gastrointestinal cancer patients [70].

#### Functional capacity

Functional capacity will be assessed using the 30-s sit-to-stand, timed-up-and-go (TUG) with cognitive task test, the four-square step test, the Berg Balance Scale, the 4-m usual walk and the 400-m walk. The 30-s sit-to-stand, four-square step test and 4-m usual walk will be performed in triplicate, with the best result recorded. All other tests will be performed once.

The 30-s sit-to-stand test measures lower-limb muscle strength and function [71]. Participants begin in a seated position with their arms folded across their chest on a chair without arms. Participants are instructed to stand fully upright and then return to a seated position as many times as possible in 30 s. This test was observed to have a test-retest ICC of 0.84 in a sample of community-dwelling men aged 60 years and over [71].

The TUG with cognitive task test is a measure of dynamic balance and dual-tasking [72]. Participants will be seated in a chair (height 45 cm) that will be placed at the end of a 3-m walkway. Once instructed to begin, the participant will stand up, walk at a regular pace for 3 m, turn, walk back to the chair and sit down. During this task, the participant will be provided with a mental arithmetic task (e.g. counting backwards from 100 by 3’s from a random number) and the number of correct digits will be recorded. A stopwatch will be used to record the time taken (to the nearest ms) to complete each test. This test was observed to have an interrater ICC of 0.99 [73].

The four-square step test is a measure of dynamic balance and stepping speed in four directions [74]. Participants will be instructed to step forward, sideways, and backwards over four canes resting flat on the floor in a cross formation in a clockwise sequence and then back to in an anticlockwise sequence. The participant will be instructed to complete the test as fast as possible without touching the raised canes, and to face forward during the entire sequence. They will also be instructed to ensure that both feet make contact with the floor in each square. The time (to the nearest ms) taken to complete the sequence will be measured with a stopwatch. This test has been observed to have an interrater and test-retest ICC of 0.99 and 0.98, respectively, in a sample of community-dwelling adults aged 65 years and over [74].

The Berg Balance Scale is an overall measure of balance including both dynamic and static components [75]. The test rates participants on 14 different tasks on an ordinal scale ranging from zero (unable to perform the task) to 4 (performs the task independently). Assessments include variations of sitting, standing, reaching, leaning, turning and stepping. The scores of the 14 tasks are summed to produce a total score ranging from 0 to 56 points. Higher scores indicate better performance. This test was observed to have an interrater and test-retest ICC of 0.98 and 0.97, respectively, in a sample of adults aged 65 years and over [75].

The 4-m usual walk is a measure of gait speed [76]. Participants will be instructed to walk at a usual pace between two cones 8 m apart (2-m acceleration zone, 4-m timed zone and 2-m deceleration zone). Time will be recorded when the participant’s front foot enters the timed zone and stopped when their front foot exits the timed zone. The time (to the nearest ms) to complete the timed zone was recorded with a stopwatch. This method has an ICC of 0.96 within our laboratory.

The 400-m walk test provides an estimate of cardiorespiratory fitness [77]. Participants will be instructed to walk as fast as possible between two cones placed 20 m apart (turning when they approach each cone). The time (to the nearest ms) taken to complete 10 laps (400 m) will be recorded. This test was observed to have a test-retest ICC of 0.95 in a sample of middle-aged women [78].

#### Biochemical, lipid and lipoprotein measures

Fasted, resting, morning blood samples will be collected at a commercial pathology clinic and sent to a central laboratory accredited by the National Association of Testing Authorities Royal College of Pathologists Australasia. The following parameters will be assessed using standardised techniques: creatinine, albumin, inorganic phosphate and corrected calcium. Fasting plasma glucose will be assessed using the hexokinase method (Roche Diagnostics, Mannheim, Germany). Total cholesterol, high-density lipoprotein cholesterol and triglycerides will be determined using an enzymatic colorimetric method (Roche Diagnostics, Mannheim, Germany). Low-density lipoprotein cholesterol will be calculated using Friedewald’s formula. The estimated glomerular filtration rate (eGFR) will be calculated using the participants’ serum creatinine, age and sex according to the abbreviated ‘modification of diet in renal disease’ formula, which is now used by most laboratories in Australia:

$$ \mathrm{eGFR}\left[\mathrm{ml}/\left(\min\;1.73\;{\mathrm{m}}^2\right)\right]=175\times {\left[\mathrm{serum}\  \mathrm{creatinine}\left(\upmu \mathrm{mol}/\mathrm{l}\right)\times 0.0113\right]}^{-1.154}\times \mathrm{age}{\left(\mathrm{years}\right)}^{-0.203}. $$

High-sensitivity C-reactive protein (CRP) will be assessed using an immunoturbidimetric assay (Roche Diagnostics, Mannheim, Germany). Total prostate-specific antigen (free and complexed) will be assessed via immunoassay (Roche Diagnostics, Mannheim, Germany).

Serum aliquots will also be collected and stored at − 80 °C so that the following parameters can be assessed in a single batch at the completion of the study if sufficient funding is obtained: serum insulin, serum 25-hydroxyvitamin D (25(OH)D), IGF-1, serum adiponectin and resistin, and a battery of pro-inflammatory and anti-inflammatory cytokines, including interleukin (IL)-6, IL-1β, IL-8, tumour necrosis factor (TNF)-α and IL-10.

#### Blood pressure

Blood pressure will be assessed three times using an automated machine (TM-2655P, A&D, Tokyo, Japan) [79] after a 10-min rest, with 1-min intervals between trials. The mean of the second and third measures will be used.

#### Cognitive function

Previous research has shown that ADT use is associated with cognitive decline, particularly in the domains of verbal memory, visuomotor function, attention and executive function [80]. Thus, this study will include a number of established and valid cognitive tests to assess the different cognitive domains. The CogState Brief Battery computerised test (http://cogstate.com/) will be administered to assess cognitive function including psychomotor function, attention, learning, reaction time, processing speed, visual attention and working memory [81, 82]. The CogState battery involves five computerised tests (Groton maze, detection task, identification task, one card learning task, one back task) conducted in a quiet environment, with scripted instructions and a practice attempt prior to completing each task.

The Rey Auditory Verbal Learning Test will be administered to assess memory and learning [83]. A list of 15 common words is read to the participant five times, with the participant asked to immediately recall as many words as possible following each recital. An interference list is then presented, after which the participant must recall words from the original list. Following a 20-min delay, during which other non-verbal tests are conducted, the participant is asked to recall the original list again. Finally, the participant will be asked to visually identify the words from the original list from a list of 50 words. Participants are scored on the total number of words recalled in trials 1–5, total number recalled after interference, loss after interference number (trial 5 minus trial 2) and long-delay free recall.

The Trail Making Test (Parts A and B) will be administered to assess executive function [84]. Participants will be instructed to accurately draw lines to connect circled numbers in sequential order as fast as possible (Part A). Participants will then be instructed to draw lines to connect circled numbers and letters in an alternating numeric and alphabetic order (Part B).

The Digit Span Test measures working memory and will be administered forwards and backwards to assess short-term memory and working memory, respectively [85]. In the first condition, the researcher presents the participant with a string of non-sequential numbers which gradually increase in length, which the participant must then immediately recall in the same order in which they were presented. In the second condition, the researcher also presents the participant with a string of non-sequential numbers which gradually increase in length, but the participant must immediately recall them in the reverse order to which they were presented.

The National Adult Reading Test will be administered to calculate verbal, performance and full-scale intelligence quotes as an estimate of cognitive reserve [86]. The test comprises of 50 irregular words which are read aloud by the participant. The number of errors regarding pronunciation are recorded.

#### Health-related quality of life, fatigue and mood

The Functional Assessment of Cancer Therapy-Fatigue (FACT-F) [87] and the Functional Assessment of Cancer Therapy-Prostate (FACT-P) [88] questionnaires will be used to assess fatigue and HR-QoL, respectively. The Assessment of Quality of Life 8D will also be used to assess HR-QoL [89]. The Geriatric Depression Scale will be used to assess depression [90]. The Depression Anxiety Stress Scale 21-item will be used to assess depression, anxiety, stress and psychological distress [91].

#### Anthropometry

Height and body mass will be assessed using a portable stadiometer and scales, respectively. Body Mass Index will be calculated using height and body mass. Girth measurements of the chest, waist and hips will be assessed using standard techniques.

#### Habitual physical activity

The Community Healthy Activities Model Programme for Seniors physical activity questionnaire will be used to assess participation in a comprehensive list of low, moderate and vigorous physical activities [92]. Participants will document the frequency and duration of their participation in a ‘typical week’ of the preceding 4 weeks. The results will be reported as estimated kilojoules per week spent in moderate- to high-intensity activities and hours per week spent undertaking weight-bearing exercise.

#### Diet

Diet will be assessed using a 24-h food recall performed during a one-on-one interview via telephone. Prior to the telephone interview, the participant will complete a 24-h food diary with instructions that will be discussed at the baseline testing session. These instructions will include how to use household measures (e.g. measuring cups, plates, bowls, glasses) to help estimate food portion sizes. In addition, a standard script will be used during the telephone interview to maximise the ability of participants to recall what was consumed. Dietary analysis will be performed using Australia-specific dietary analysis software (FoodWorks, Xyris software, Highgate Hills, QLD, Australia).

#### Additional questionnaires

A demographic, clinical and lifestyle questionnaire will be used to obtain background information from participants (e.g. age, education history, marital status, employment status, cultural background, PCa and ADT-use details). Sun-exposure habits and practices, alcohol consumption, past and current medical conditions, use of prescription and non-prescription medication (type and dose) and falls and fracture history will also be included in the questionnaire.

#### Compliance

Compliance with the exercise training programme (i.e. attendance at each training session) and nutritional supplement (i.e. consumption) will be assessed via attendance record and self-reported diaries, respectively. Diaries will be monitored weekly and calendars will be monitored monthly by the participant’s accredited exercise physiologist and researchers. In addition to the calendars, compliance with the sachets and vitamin D tablets will be checked by counting all returned at the 26- and 52-week testing sessions.

#### Adverse events

Any adverse events potentially associated, or confirmed to be associated, with the exercise programme or protein, calcium and vitamin D supplementation will be recorded by the exercise trainers at each exercise training session for the intervention-group participants and monthly telephone calls by the research staff to the control-group participants. For this trial, an adverse event will consist of any health-related unfavourable or unintended medical occurrence (e.g. sign, symptom, syndrome, illness) that develops or worsens during the trial. All adverse events will be assessed for seriousness, causality and expected outcome by the researchers and followed during the trial. Although there will be no formal data monitoring committee, all data will be reviewed at regular intervals throughout the study and researchers involved in recruitment, testing and implementing the trial will update the research team monthly about the study progress.

#### Data management and archiving

All data will be stored on password-protected computers and in locked filing cabinets at Deakin University (Burwood). All study-related documents will be archived at Deakin University at the end of the study for 7 years which is in line with current ethical requirements.

#### Dissemination plan

Findings from the primary outcomes of this trial will be reported in journal articles, which will include results regardless of the direction or magnitude of the effect. The results will also be presented at leading national and international conferences, clinical forums and to other relevant health professionals and stakeholders, as well as to the participants. All investigators will have the opportunity to be listed as an author of future publications in accordance with journal-specific guidelines.

### Sample size calculation

The sample size calculations are based on our previous research in healthy and ADT-treated men. The mean (standard deviation (SD)) annual loss in femoral neck, hip and spine BMD in ADT-treated men was 3.0% (4.5), 2.6% (2.8) and 3.9% (3.0), respectively [14]. Our previous exercise trials in healthy older men have shown that 12 months of training can increase spine aBMD by 1.5–2.0% (SD 2.9) and hip aBMD by 1.2–1.8% (SD 2.8) [39, 55]. We estimate a conservative net difference in aBMD in favour of the intervention of ~ 3.5–4.0% at the hip and spine. Based on these estimates, 29 men in each group will provide 90% power (*P* < 0.05, two-tailed) to detect a difference of this magnitude (assuming a SD of 4.0). A net benefit of this magnitude is clinically relevant as there is evidence that a 1–2% gain in aBMD translates into a 7–14% reduction in fracture risk [36, 93]. For pQCT bone measures, radial and tibial cortical area decreased by 11.5% (8.8) and 12.5% (7.4), respectively, over 12 months in ADT-treated men [14]. While few long-term trials have examined the effect of exercise training on pQCT bone geometry in older men, several intervention trials in older adults have shown that a targeted, weight-bearing, impact-exercise programme can have beneficial effects on cortical bone [94, 95]. Assuming that the proposed intervention can half the rate of loss in cortical area, we estimate that we will require 39 men per group (SD 8.0; 90% power, two-tailed, *P* < 0.05). Based on a previous study and a meta-analysis showing that ADT is associated with an average 7.7% gain in fat and a 2.8% loss in lean mass over 3–12 months [96, 97], and that exercise training can prevent the gain in FM and increase (1–5%) or preserve LBM relative to usual care in men on ADT [30, 32], we estimate a sample size of 24–32 in each group will be required to observe a preservation in LBM and FM in the intervention group vs. usual care (90% power, two-tailed, *P* < 0.05). Assuming a conservatively projected 30% dropout, an estimated sample size of 51 men per group (102 in total) will be required. Based on previous studies in ADT-treated men focussing on a number of our secondary outcomes [32, 37, 98], we estimate that we will have sufficient power (80–90%, *P* < 0.05, two-tailed) to detect the following intervention effects: 20–40% for muscle strength; 8–22% for functional capacity; 4 points for fatigue (FACT-F), 15% for fasting plasma glucose and 14% for serum IL-6 and 20% for CRP. We will also have sufficient sample to detect a clinically meaningful difference of 0.5 SD in the Assessment of Quality of Life 8D questionnaire (AQoL-8D) and 9 points on the FACT-P (disease-specific QoL).

### Statistical analysis

Primary analyses will be conducted on an intention-to-treat basis using STATA statistical software (STATA, College Station, TX, USA). Per-protocol analysis will also be performed by including all participants who are at least 66% compliant to the exercise (two of three sessions per week) and 80% compliant to taking the supplements. Initially, descriptive statistics will be computed to compare the intervention and usual-care groups on background variables and baseline measures. All data will be screened for outliers and the normality of the distribution of all data will be assessed using Kolmogorov-Smirnov tests, with skewed data log transformed prior to analysis. Linear mixed models with random effects will be used to evaluate the effects of the intervention compared to usual control. Potential covariates to be included will be: age, duration of ADT use, other chronic conditions, change in medication and change in habitual physical activity or diet. Where possible, we will obtain endpoint measures from all withdrawals and include all randomised subjects in the final analysis. For participants who are lost to follow-up, missing data will be handled with multiple imputation, with subsequent sensitivity analyses to evaluate the effect of potential non-random attrition. Baseline measures and changes in outcome variables will be presented as means ± SD or 95% confidence intervals. A significance level of *P* < 0.05 will be adopted for all statistical tests.

## Discussion

This trial will be the first to assess the efficacy of combining a targeted, bone-specific, resistance and impact exercise training programme with nutritional supplementation on musculoskeletal health in men treated with ADT for PCa. Importantly, this is one of the few known trials to examine such a lifestyle intervention over an extended period of time (52 weeks) in this population group, with a focus on both DXA areal bone density and pQCT measures of bone structure and cortical and trabecular volumetric BMD, all of which are important determinants of whole bone strength. There are currently no established guidelines for specifically managing the large range of adverse effects observed in men treated with ADT [28]. Current guidelines mainly focus on pharmacological interventions for bone health, such as antiresorptive therapy with bisphosphonates, which have been extensively evaluated and shown to prevent losses in bone density commonly reported with ADT [11, 99–101]. However, these drugs have no effect on ameliorating the many other adverse effects associated with ADT. In terms of muscle health, functional capacity and cardiometabolic risk, the guidelines are less evidence-based within this specific clinical population group [11, 99], and commonly recommend more generic exercise guidelines for cancer survivors [102–105]. A recent study evaluating the most robust ADT-specific guidelines [11] in a cohort of 113 men commencing ADT concluded that over a 2-year follow-up, despite maintaining bone density at the lumbar spine and reducing various cardiometabolic risk markers via additional therapies (e.g. statins), bone losses were still reported at the total hip, and risk factors of cardiometabolic risk, such as waist circumference, increased. Therefore, further well-designed, long-term trials are needed to inform ADT-specific exercise training guidelines for managing bone health and cardiometabolic risk. Furthermore, there are limited guidelines on addressing declines in HR-QoL and the large range of additional ADT-induced adverse effects. We expect that the findings from this study will provide a unique opportunity to explore whether combining exercise training with nutritional supplementation may also be effective at ameliorating multiple ADT-induced adverse effects when compared to usual care.

### Trial status

The IMPACT trial is concurrently recruiting and administering the intervention to a number of participants.

## Additional files

Additional file 1: Trial registration data. (DOCX 15 kb)

Additional file 2: SPIRIT checklist. (DOC 120 kb)

## Abbreviations

3RM: Three-repetition maximum
aBMD: Areal bone mineral density
ADT: Androgen deprivation therapy
BSI: Bone Strength Index
CRP: C-reactive protein
CV: Coefficient of variation
DXA: Dual-energy X-ray absorptiometry
eGFR: Estimated glomerular filtration rate
FACT-F: Functional Assessment of Cancer Therapy-Fatigue
FACT-P: Functional Assessment of Cancer Therapy-Prostate
FM: Fat mass
GnRH: Gonadotropin-releasing hormone
HR-QoL: Health-related quality of life
ICC: Intraclass correlation coefficient
IGF-1: Serum insulin-like growth factor-1
IL: Interleukin
LBM: Lean body mass
MPB: Muscle protein breakdown
MPS: Muscle protein synthesis
PCa: Prostate cancer
pQCT: Peripheral quantitative computed tomography
PRT: Progressive resistance training
RCT: Randomised controlled trial
RPE: Rating of Perceived Exertion
SSI: Stress Strain Index
TNF: Tumour necrosis factor
TUG: Timed-up-and-go
vBMD: Volumetric bone mineral density

## References

[1] Torre LA, Bray F, Siegel RL, Ferlay J, Lortet-Tieulent J, Jemal A (2015). Global cancer statistics, 2012. CA Cancer J Clin.

[2] Australian Institute of Health and Welfare (2014). Cancer in Australia: an overview 2014.

[3] DeSantis CE, Lin CC, Mariotto AB, Siegel RL, Stein KD, Kramer JL, Alteri R, Robbins AS, Jemal A (2014). Cancer treatment and survivorship statistics, 2014. CA Cancer J Clin.

[4] Bolla M, Collette L, Blank L, Warde P, Dubois JB, Mirimanoff R-O, Storme G, Bernier J, Kuten A, Sternberg C (2002). Long-term results with immediate androgen suppression and external irradiation in patients with locally advanced prostate cancer (an EORTC study): a phase III randomised trial. Lancet.

[5] Denham JW, Steigler A, Lamb DS, Joseph D, Mameghan H, Turner S, Matthews J, Franklin I, Atkinson C, North J (2005). Short-term androgen deprivation and radiotherapy for locally advanced prostate cancer: results from the Trans-Tasman Radiation Oncology Group 96.01 randomised controlled trial. Lancet Oncol.

[6] Messing EM, Manola J, Yao J, Kiernan M, Crawford D, Wilding G, di’SantAgnese PA, Trump D (2006). Immediate versus deferred androgen deprivation treatment in patients with node-positive prostate cancer after radical prostatectomy and pelvic lymphadenectomy. Lancet Oncol.

[7] Pilepich MV, Winter K, Lawton CA, Krisch RE, Wolkov HB, Movsas B, Hug EB, Asbell SO, Grignon D (2005). Androgen suppression adjuvant to definitive radiotherapy in prostate carcinoma: long-term results of phase III RTOG 85–31. Int J Radiat Oncol Biol Phys.

[8] D’Amico AV, Manola J, Loffredo M, Renshaw AA, DellaCroce A, Kantoff PW (2004). 6-month androgen suppression plus radiation therapy vs radiation therapy alone for patients with clinically localized prostate cancer: a randomized controlled trial. JAMA.

[9] Lu-Yao GL, Albertsen PC, Moore DF, Shih W, Lin Y, DiPaola R, Yao S-L (2008). Survival following primary androgen deprivation therapy among men with localized prostate cancer. JAMA.

[10] Shahinian VB, Kuo YF, Freeman JL, Orihuela E, Goodwin JS (2005). Increasing use of gonadotropin-releasing hormone agonists for the treatment of localized prostate carcinoma. Cancer.

[11] Grossmann M, Hamilton EJ, Gilfillan C, Bolton D, Joon DL, Zajac JD (2011). Bone and metabolic health in patients with non-metastatic prostate cancer who are receiving androgen deprivation therapy. Med J Aust.

[12] Medicare Australia. Pharmaceutical Benefits Schedule item reports. http://medicarestatistics.humanservices.gov.au/statistics/pbs_item.jsp. Accessed 21 Mar 2016.

[13] Cheung AS, Zajac JD, Grossmann M (2014). Muscle and bone effects of androgen deprivation; current and emerging therapies. Endocr Relat Cancer.

[14] Hamilton EJ, Ghasem-Zadeh A, Gianatti E, Lim-Joon D, Bolton D, Zebaze R, Seeman E, Zajac JD, Grossmann M (2010). Structural decay of bone microarchitecture in men with prostate cancer treated with androgen deprivation therapy. J Clin Endocrinol Metab.

[15] Spry NA, Taaffe DR, England PJ, Judge JS, Stephens DA, Peddle-McIntyre C, Baker MK, Newton RU, Galvão DA (2013). Long-term effects of intermittent androgen suppression therapy on lean and fat mass: a 33-month prospective study. Prostate Cancer Prostatic Dis.

[16] Bolam KA, Galvão DA, Spry N, Newton RU, Taaffe DR (2012). AST-induced bone loss in men with prostate cancer: exercise as a potential countermeasure. Prostate Cancer Prostatic Dis.

[17] Owen PJ, Daly RM, Livingston PM, Fraser SF (2017). Lifestyle guidelines for managing adverse effects on bone health and body composition in men treated with androgen deprivation therapy for prostate cancer: an update. Prostate Cancer Prostatic Dis.

[18] Alibhai SMH, Duong-Hua M, Cheung AM, Sutradhar R, Warde P, Fleshner NE, Paszat L (2010). Fracture types and risk factors in men with prostate cancer on androgen deprivation therapy: a matched cohort study of 19,079 men. J Urol.

[19] Beebe-Dimmer JL, Cetin K, Shahinian V, Morgenstern H, Yee C, Schwartz KL, Acquavella J (2012). Timing of androgen deprivation therapy use and fracture risk among elderly men with prostate cancer in the United States. Pharmacoepidemiol Drug Saf.

[20] Shao Y-H, Moore DF, Shih W, Lin Y, Jang TL, Lu-Yao GL (2013). Fracture after androgen deprivation therapy among men with a high baseline risk of skeletal complications. BJU Int.

[21] Cheung AS, Pattison D, Bretherton I, Hoermann R, Lim Joon D, Ho E, Jenkins T, Hamilton EJ, Bate K, Chan I (2013). Cardiovascular risk and bone loss in men undergoing androgen deprivation therapy for non-metastatic prostate cancer: implementation of standardized management guidelines. Andrology.

[22] Grossmann M, Cheung AS, Zajac JD (2013). Androgens and prostate cancer; pathogenesis and deprivation therapy. Best Pract Res Cl En.

[23] Keating NL, O’Malley AJ, Smith MR (2006). Diabetes and cardiovascular disease during androgen deprivation therapy for prostate cancer. J Clin Oncol.

[24] Lage MJ, Barber BL, Markus RA (2007). Association between androgen-deprivation therapy and incidence of diabetes among males with prostate cancer. Urology.

[25] Saigal CS, Gore JL, Krupski TL, Hanley J, Schonlau M, Litwin MS (2007). Androgen deprivation therapy increases cardiovascular morbidity in men with prostate cancer. Cancer.

[26] Gardner JR, Livingston PM, Fraser SF (2014). Effects of exercise on treatment-related adverse effects for patients with prostate cancer receiving androgen-deprivation therapy: a systematic review. J Clin Oncol.

[27] Newton R, Galvão D (2013). Exercise medicine for prostate cancer. Eur Rev Aging Phys Act.

[28] Owen PJ, Fraser SF (2015). The role of exercise training in men with prostate cancer. Top Geriatr Rehabil.

[29] Bourke L, Doll H, Crank H, Daley A, Rosario D, Saxton JM (2011). Lifestyle intervention in men with advanced prostate cancer receiving androgen suppression therapy: a feasibility study. Cancer Epidemiol Biomarkers Prev.

[30] Galvão DA, Taaffe DR, Spry N, Joseph D, Newton RU (2010). Combined resistance and aerobic exercise program reverses muscle loss in men undergoing androgen suppression therapy for prostate cancer without bone metastases: a randomized controlled trial. J Clin Oncol.

[31] Segal RJ, Reid RD, Courneya KS, Sigal RJ, Kenny GP, Prud’Homme DG, Malone SC, Wells GA, Scott CG, Slovinec D’Angelo ME (2009). Randomized controlled trial of resistance or aerobic exercise in men receiving radiation therapy for prostate cancer. J Clin Oncol.

[32] Cormie P, Galvão DA, Spry N, Joseph D, Chee R, Taaffe DR, Chambers SK, Newton RU (2015). Can supervised exercise prevent treatment toxicity in patients with prostate cancer initiating androgen-deprivation therapy: a randomised controlled trial. BJU Int.

[33] Nilsen TS, Raastad T, Skovlund E, Courneya KS, Langberg CW, Lilleby W, Fosså SD, Thorsen L (2015). Effects of strength training on body composition, physical functioning, and quality of life in prostate cancer patients during androgen deprivation therapy. Acta Oncol.

[34] Segal RJ, Reid RD, Courneya KS, Malone SC, Parliament MB, Scott CG, Venner PM, Quinney HA, Jones LW, Slovinec D’Angelo ME, Wells GA (2003). Resistance exercise in men receiving androgen deprivation therapy for prostate cancer. J Clin Oncol.

[35] Alberga A, Segal R, Reid R, Scott C, Sigal R, Khandwala F, Jaffey J, Wells G, Kenny G (2012). Age and androgen-deprivation therapy on exercise outcomes in men with prostate cancer. Support Care Cancer.

[36] Winters-Stone KM, Dobek JC, Bennett JA, Maddalozzo GF, Ryan CW, Beer TM (2014). Skeletal response to resistance and impact training in prostate cancer survivors. Med Sci Sports Exerc.

[37] Galvão DA, Nosaka K, Taaffe DR, Spry N, Kristjanson LJ, McGuigan MR, Suzuki K, Yamaya K, Newton RU (2006). Resistance training and reduction of treatment side effects in prostate cancer patients. Med Sci Sport Exerc.

[38] Daly R, Duckham R, Gianoudis J (2014). Evidence for an interaction between exercise and nutrition for improving bone and muscle health. Curr Osteoporos Rep.

[39] Kukuljan S, Nowson CA, Sanders KM, Nicholson GC, Seibel MJ, Salmon J, Daly RM (2011). Independent and combined effects of calcium-vitamin D3 and exercise on bone structure and strength in older men: an 18-month factorial design randomized controlled trial. J Clin Endocrinol Metab.

[40] Datta M, Schwartz GG (2012). Calcium and vitamin D supplementation during androgen deprivation therapy for prostate cancer: a critical review. Oncologist.

[41] Nowson CA, McGrath JJ, Ebeling PR, Haikerwal A, Daly RM, Sanders KM, Seibel MJ, Mason RS (2012). Vitamin D and health in adults in Australia and New Zealand: a position statement. Med J Aust.

[42] Pirotta S, Kidgell DJ, Daly RM (2015). Effects of vitamin D supplementation on neuroplasticity in older adults: a double-blinded, placebo-controlled randomised trial. Osteoporos Int.

[43] Daly RM, Brown M, Bass S, Kukuljan S, Nowson C (2006). Calcium- and vitamin D3-fortified milk reduces bone loss at clinically relevant skeletal sites in older men: a 2-year randomized controlled trial. J Bone Miner Res.

[44] Aune D, Navarro Rosenblatt DA, Chan DS, Vieira AR, Vieira R, Greenwood DC, Vatten LJ, Norat T (2015). Dairy products, calcium, and prostate cancer risk: a systematic review and meta-analysis of cohort studies. Am J Clin Nutr.

[45] Greenspan SL (2008). Approach to the prostate cancer patient with bone disease. J Clin Endocrinol Metab.

[46] Bristow SM, Bolland MJ, MacLennan GS, Avenell A, Grey A, Gamble GD, Reid IR (2013). Calcium supplements and cancer risk: a meta-analysis of randomised controlled trials. Brit J Nutr.

[47] Kristal AR, Chi C, Tangen CM, Goodman PJ, Etzioni R, Thompson IM (2006). Associations of demographic and lifestyle characteristics with prostate-specific antigen (PSA) concentration and rate of PSA increase. Cancer.

[48] Pettersson A, Kasperzyk JL, Kenfield SA, Richman EL, Chan JM, Willett WC, Stampfer MJ, Mucci LA, Giovannucci EL (2012). Milk and dairy consumption among men with prostate cancer and risk of metastases and prostate cancer death. Cancer Epidem Biomar.

[49] Calvez J, Poupin N, Chesneau C, Lassale C, Tome D (2012). Protein intake, calcium balance and health consequences. Eur J Clin Nutr.

[50] Pal S, Radavelli-Bagatini S (2013). The effects of whey protein on cardiometabolic risk factors. Obes Rev.

[51] Churchward-Venne TA, Holwerda AM, Phillips SM, van Loon LJC. What is the optimal amount of protein to support post-exercise skeletal muscle reconditioning in the older adult? Sports Med. 2016;doi:10.1007/s40279-016-0504-2. 10.1007/s40279-016-0504-2 26894275

[52] Hanson ED, Nelson AR, West DW, Violet JA, O’keefe L, Phillips SM, Hayes A (2017). Attenuation of resting but not load-mediated protein synthesis in prostate cancer patients on androgen deprivation. J Clin Endocr Metab.

[53] American College of Sports Medicine (2010). ACSM’s resource manual for guidelines for exercise testing and prescription.

[54] Gianoudis J, Bailey C, Sanders K, Nowson C, Hill K, Ebeling P, Daly R (2012). Osteo-cise: strong bones for life: protocol for a community-based randomised controlled trial of a multi-modal exercise and osteoporosis education program for older adults at risk of falls and fractures. BMC Musculoskelet Disord.

[55] Gianoudis J, Bailey CA, Ebeling PR, Nowson CA, Sanders KM, Hill K, Daly RM (2014). Effects of a targeted multimodal exercise program incorporating high-speed power training on falls and fracture risk factors in older adults: a community-based randomized controlled trial. J Bone Miner Res.

[56] Kibler WB, Press J, Sciascia A (2006). The role of core stability in athletic function. Sports Med.

[57] Mamerow MM, Mettler JA, English KL, Casperson SL, Arentson-Lantz E, Sheffield-Moore M, Layman DK, Paddon-Jones D (2014). Dietary protein distribution positively influences 24-h muscle protein synthesis in healthy adults. J Nutr.

[58] Esmarck B, Andersen JL, Olsen S, Richter EA, Mizuno M, Kjær M (2001). Timing of postexercise protein intake is important for muscle hypertrophy with resistance training in elderly humans. J Physiol.

[59] Pennings B, Koopman R, Beelen M, Senden JM, Saris WH, van Loon LJ (2011). Exercising before protein intake allows for greater use of dietary protein—derived amino acids for de novo muscle protein synthesis in both young and elderly men. Am J Clin Nutr.

[60] Doube M, Klosowski MM, Arganda-Carreras I, Cordelieres FP, Dougherty RP, Jackson JS, Schmid B, Hutchinson JR, Shefelbine SJ (2010). BoneJ: free and extensible bone image analysis in ImageJ. Bone.

[61] Rantalainen T, Nikander R, Heinonen A, Daly RM, Sievanen H (2011). An open source approach for regional cortical bone mineral density analysis. J Musculoskel Neuron.

[62] Kontulainen SA, Johnston JD, Liu D, Leung C, Oxland TR, McKay HA (2008). Strength indices from pQCT imaging predict up to 85% of variance in bone failure properties at tibial epiphysis and diaphysis. J Musculoskel Neuron.

[63] Laskey MA, de Bono S, Zhu D, Shaw CN, Laskey PJ, Ward KA, Prentice A (2010). Evidence for enhanced characterization of cortical bone using novel pQCT shape software. J Clin Densitom.

[64] Rinaldi G, Wisniewski CA, Setty NG, Leboff MS (2011). Peripheral quantitative computed tomography: optimization of reproducibility measures of bone density, geometry, and strength at the radius and tibia. J Clin Densitom.

[65] Frank-Wilson AW, Johnston JD, Olszynski WP, Kontulainen SA (2015). Measurement of muscle and fat in postmenopausal women: Precision of previously reported pQCT imaging methods. Bone.

[66] Wathen D (1994). Load assignment.

[67] McCurdy K, Langford GA, Cline AL, Doscher M, Hoff R (2004). The reliability of 1- and 3rm tests of unilateral strength in trained and untrained men and women. J Sports Sci Med.

[68] Bishop A, DeBeliso M, Sevene TG, Adams KJ (2014). Comparing one repetition maximum and three repetition maximum between conventional and eccentrically loaded deadlifts. J Strength Cond Res.

[69] Roberts HC, Denison HJ, Martin HJ, Patel HP, Syddall H, Cooper C, Sayer AA (2011). A review of the measurement of grip strength in clinical and epidemiological studies: towards a standardised approach. Age Ageing.

[70] Trutschnigg B, Kilgour RD, Reinglas J, Rosenthall L, Hornby L, Morais JA, Vigano A (2008). Precision and reliability of strength (Jamar vs. Biodex handgrip) and body composition (dual-energy X-ray absorptiometry vs. bioimpedance analysis) measurements in advanced cancer patients. Appl Physiol Nutr Me.

[71] Jones C, Rikli R, Beam W (1999). A 30-s chair-stand test as a measure of lower body strength in community-residing older adults. Res Q Exerc Sport.

[72] Tang PF, Yang HJ, Peng YC, Chen HY (2015). Motor dual-task timed up & go test better identifies prefrailty individuals than single-task timed up & go test. Geriatr Gerontol Int.

[73] Shumway-Cook A, Brauer S, Woollacott M (2000). Predicting the probability for falls in community-dwelling older adults using the timed up & go test. Phys Ther.

[74] Dite W, Temple VA (2002). A clinical test of stepping and change of direction to identify multiple falling older adults. Arch Phys Med Rehabil.

[75] Berg K (1989). Measuring balance in the elderly: preliminary development of an instrument. Physiother Can.

[76] Peters DM, Fritz SL, Krotish DE (2013). Assessing the reliability and validity of a shorter walk test compared with the 10-meter walk test for measurements of gait speed in healthy, older adults. J Geriatr Phys Ther.

[77] Simonsick EM, Fan E, Fleg JL (2006). Estimating cardiorespiratory fitness in well-functioning older adults: treadmill validation of the long distance corridor walk. J Am Geriatr Soc.

[78] Gabriel KKP, Rankin RL, Chong L, Charlton ME, Swan PD, Ainsworth BE (2010). Test-retest reliability and validity of the 400-meter walk test in healthy, middle-aged women. J Phys Act Health.

[79] Kobalava ZD, Kotovskaya YV, Babaeva LA, Moiseev VS (2006). Validation of TM-2655 oscillometric device for blood pressure measurement. Blood Press Monit.

[80] Mundell NL, Daly RM, Macpherson H, Fraser SF (2017). Cognitive decline in prostate cancer patients undergoing ADT: a potential role for exercise training. Cognitive decline in prostate cancer patients undergoing ADT: a potential role for exercise training. Endocr Relat Cancer.

[81] Darby DG, Pietrzak RH, Fredrickson J, Woodward M, Moore L, Fredrickson A, Sach J, Maruff P (2012). Intraindividual cognitive decline using a brief computerized cognitive screening test. Alzheimers Dement.

[82] Maruff P, Thomas E, Cysique L, Brew B, Collie A, Snyder P, Pietrzak RH (2009). Validity of the CogState brief battery: relationship to standardized tests and sensitivity to cognitive impairment in mild traumatic brain injury, schizophrenia, and AIDS dementia complex. Arch Clin Neuropsych.

[83] Schoenberg MR, Dawson KA, Duff K, Patton D, Scott JG, Adams RL (2006). Test performance and classification statistics for the Rey Auditory Verbal Learning Test in selected clinical samples. Arch Clin Neuropsychol.

[84] Salthouse TA (2011). What cognitive abilities are involved in trail-making performance?. Intelligence.

[85] Schroeder RW, Twumasi-Ankrah P, Baade LE, Marshall PS (2012). Reliable digit span: a systematic review and cross-validation study. Assessment.

[86] Nelson HE, Willison J (1991). The National Adult Reading Test (NART).

[87] Yellen SB, Cella DF, Webster K, Blendowski C, Kaplan E (1997). Measuring fatigue and other anemia-related symptoms with the Functional Assessment of Cancer Therapy (FACT) measurement system. J Pain Symptom Manag.

[88] Esper P, Mo F, Chodak G, Sinner M, Cella D, Pienta KJ (1997). Measuring quality of life in men with prostate cancer using the Functional Assessment of Cancer Therapy-Prostate instrument. Urology.

[89] Richardson J, Sinha K, Iezzi A, Khan MA (2014). Modelling utility weights for the Assessment of Quality of Life (AQoL)-8D. Qual Life Res.

[90] Burke WJ, Roccaforte WH, Wengel SP (1991). The short form of the Geriatric Depression Scale: a comparison with the 30-item form. J Geriatr Psych Neur.

[91] Antony MM, Bieling PJ, Cox BJ, Enns MW, Swinson RP (1998). Psychometric properties of the 42-item and 21-item versions of the Depression Anxiety Stress Scales in clinical groups and a community sample. Psychol Assessment.

[92] Stewart AL, Mills KM, King AC, Haskell WL, Gillis D, Ritter PL (2001). CHAMPS physical activity questionnaire for older adults: outcomes for interventions. Med Sci Sports Exerc.

[93] Kelley GA, Kelley KS, Kohrt WM (2012). Effects of ground and joint reaction force exercise on lumbar spine and femoral neck bone mineral density in postmenopausal women: a meta-analysis of randomized controlled trials. BMC Musculoskelet Disord.

[94] Adami S, Gatti D, Braga V, Bianchini D, Rossini M (1999). Site-specific effects of strength training on bone structure and geometry of ultradistal radius in postmenopausal women. J Bone Miner Res.

[95] Allison SJ, Poole KES, Treece GM, Gee AH, Tonkin C, Rennie WJ, Folland JP, Summers GD, Brooke-Wavell K (2015). The influence of high-impact exercise on cortical and trabecular bone mineral content and 3D distribution across the proximal femur in older men: a randomized controlled unilateral intervention. J Bone Miner Res.

[96] Haseen F, Murray L, O’Neill R, O’Sullivan J, Cantwell M (2010). A randomised controlled trial to evaluate the efficacy of a 6 month dietary and physical activity intervention for prostate cancer patients receiving androgen deprivation therapy. Trials.

[97] Spry NA, Galvão DA, Davies R, La Bianca S, Joseph D, Davidson A, Prince R (2009). Long-term effects of intermittent androgen suppression on testosterone recovery and bone mineral density: results of a 33-month observational study. BJU Int.

[98] Galvao DA, Taaffe DR, Cormie P, Spry N, Joseph DJ, Chambers SK, Gardiner RA, Bolam K, Wall BA, Newton RU (2014). A multicenter yearlong randomized controlled trial of different exercise modalities in prostate cancer survivors on androgen deprivation therapy. J Clin Oncol.

[99] Saylor P, Keating N, Smith M (2009). Prostate cancer survivorship: prevention and treatment of the adverse effects of androgen deprivation therapy. J Gen Intern Med.

[100] Lee CE, Leslie WD, Czaykowski P, Gingerich J, Geirnaert M, Lau YKJ (2011). A comprehensive bone-health management approach for men with prostate cancer receiving androgen deprivation therapy. Curr Oncol.

[101] Graham J, Kirkbride P, Cann K, Hasler E, Prettyjohns M (2014). Prostate cancer: summary of updated NICE guidance. BMJ.

[102] Campbell A, Stevinson C, Crank H (2012). The BASES expert statement on exercise and cancer survivorship. J Sport Sci.

[103] Hayes SC, Spence RR, Galvão DA, Newton RU (2009). Australian Association for Exercise and Sport Science position stand: optimising cancer outcomes through exercise. J Sci Med Sport.

[104] Rock CL, Doyle C, Demark-Wahnefried W, Meyerhardt J, Courneya KS, Schwartz AL, Bandera EV, Hamilton KK, Grant B, McCullough M (2012). Nutrition and physical activity guidelines for cancer survivors. CA Cancer J Clin.

[105] Schmitz KH, Courneya KS, Matthews C, Demark-Wahnefried W, Galvão DA, Pinto BM, Irwin ML, Wolin KY, Segal RJ, Lucia A (2010). American College of Sports Medicine roundtable on exercise guidelines for cancer survivors. Med Sci Sport Exerc.

