# Supplementary material for: Efficacy of a multi-component exercise programme and nutritional supplementation on musculoskeletal health in men treated with androgen deprivation therapy for prostate cancer (IMPACT): study protocol of a randomised controlled trial
Source: Trials. 2017 Oct 3;18:451. doi: 10.1186/s13063-017-2185-z (PMC5627417; doi:10.1186/s13063-017-2185-z)
Supplement: Supplementary file 1 — Trial registration data. (DOCX 15 kb) [file 13063_2017_2185_MOESM1_ESM.docx]

**Additional file 1.** Summary of trial registration data

| **Data category** | **Information** |
| --- | --- |
| Primary registry and trial identifying number | Australian and New Zealand Clinical Trials Registry (ACTRN12614000317695) |
| Date of registration in primary registry | 25 March, 2014 |
| Secondary identifying numbers | Deakin University (HREC 2013-184); Alfred Health (Project No: 455/15) |
| Source(s) of monetary or material support | Deakin University |
| Primary sponsor | Deakin University |
| Secondary sponsor(s) | YMCA Victoria; Ostelin; Omniblend |
| Contact for public queries | Dr Steve Fraser [steve.fraser@deakin.edu.au] |
| Contact for scientific queries | Dr Steve Fraser [steve.fraser@deakin.edu.au] |
| Public title | The efficacy of exercise training and nutritional supplementation in prostate cancer survivors |
| Scientific title | The efficacy of resistance exercise training and nutritional supplementation of calcium, vitamin D and protein on musculoskeletal health, cardiometabolic risk factors and health-related quality of life in prostate cancer survivors treated with androgen deprivation therapy when compared to usual care |
| Countries of recruitment | Australia |
| Health condition(s) or problem(s) studied | Prostate cancer survivors treated with androgen deprivation therapy |
| Intervention(s) | Intervention: Multi-component resistance and impact loading exercise program and a daily protein (25 g), calcium (~480 mg elemental) and vitamin D (2000 IU) enriched supplement |
|  | Control: Usual care (including 1000 IU/d vitamin D) |
| Key inclusion and exclusion criteria | Ages eligible for study: 50-85 years; Sexes eligible for study: Male; Accepts healthy volunteers: No |
|  | Inclusion criteria: Men aged 50-85 year currently treated with pharmacological androgen deprivation therapy for histologically diagnosed prostate cancer for greater than 12 weeks |
|  | Exclusion criteria: do not have the ability to complete surveys in the English language; any disorder known to affect bone, calcium or vitamin D metabolism (other than hypogonadism); currently receiving pharmacological intervention known to affect bone metabolism (other than androgen deprivation therapy); supplementation with protein, calcium (>600 mg/d) or vitamin D (>1000 IU/d) in the past three months; have undertaken progressive resistance training (>1 session/wk) or high impact weight bearing exercise (>150 min/wk) in the past three months; current smokers; weight greater than 159kg; have plans to travel for greater than six weeks continuously within the following 52 weeks; any absolute contraindications to exercise training according to the American College of Sports Medicine guidelines |
| Study type | Interventional |
|  | Allocation: randomised; Intervention model; parallel assignment; Masking: Open (masking not used) |
|  | Primary purpose: Treatment |
|  | Phase IV |
| Date of first enrolment | August, 2014 |
| Target sample size | 102 |
| Recruitment status | Recruiting |
| Primary outcome(s) | Areal bone mineral density |
| Key secondary outcomes | Bone structure and strength; Body composition; Muscle strength; Functional capacity; Biochemistry and lipids; Inflammatory markers; Blood pressure; Cognitive function; Health-related quality of life, fatigue and mood |
